# Grf10 and Bas1 Regulate Transcription of Adenylate and One-Carbon Biosynthesis Genes and Affect Virulence in the Human Fungal Pathogen *Candida albicans*

**DOI:** 10.1128/mSphere.00161-17

**Published:** 2017-08-02

**Authors:** Tanaporn Wangsanut, Anup K. Ghosh, Peter G. Metzger, William A. Fonzi, Ronda J. Rolfes

**Affiliations:** a Department of Biology, Georgetown University, Washington, DC, USA; b Department of Microbiology and Immunology, Georgetown University, Washington, DC, USA; University College Dublin, Belfield

**Keywords:** Bas1, *Candida albicans*, Grf10, fungal pathogen, one-carbon metabolism, purine metabolism, transcriptional regulation, virulence

## Abstract

*Candida albicans* is an opportunistic human fungal pathogen that causes superficial fungal infections and lethal systemic infections. To colonize and establish infections, *C. albicans* coordinates the expression of virulence and metabolic genes. Previous work showed that the homeodomain transcription factor Grf10 is required for formation of hyphae, a virulence factor. Here we report global gene expression analysis of a *grf10*Δ strain using a DNA microarray and identify genes for *de novo* adenylate biosynthesis (*ADE* genes), one-carbon metabolism, and a nucleoside permease (*NUP*). Upregulation of these genes in response to adenine limitation required both Grf10 and the *myb* protein Bas1, as shown by quantitative real-time PCR (qRT-PCR). Phenotypic analysis showed that both mutants exhibited growth defects when grown in the absence of adenine, and the doubling time was slower for the *bas1*Δ mutant. Bas1 is required for basal expression of these genes, whereas *NUP* expression is more dependent upon Grf10. Disruption of *BAS1* led to only modest defects in hypha formation and weak attenuation of virulence in a systemic mouse model of infection, as opposed to the previously reported strong effects found in the *grf10*Δ mutant. Our data are consistent with a model in which Grf10 coordinates metabolic effects on nucleotide metabolism by interaction with Bas1 and indicate that AMP biosynthesis and its regulation are important for *C. albicans* growth and virulence.

**IMPORTANCE** *Candida albicans* is a commensal and a common constituent of the human microbiota; however, it can become pathogenic and cause infections in both immunocompetent and immunocompromised people. *C. albicans* exhibits remarkable metabolic versatility as it can colonize multiple body sites as a commensal or pathogen. Understanding how *C. albicans* adapts metabolically to each ecological niche is essential for developing novel therapeutic approaches. Purine metabolism has been targeted pharmaceutically in several diseases; however, the regulation of this pathway has not been fully elucidated in *C. albicans*. Here, we report how *C. albicans* controls the AMP *de novo* biosynthesis pathway in response to purine availability. We show that the lack of the transcription factors Grf10 and Bas1 leads to purine metabolic dysfunction, and this dysfunction affects the ability of *C. albicans* to establish infections.

## Abstract (precis)

*Candida albicans* is a commensal and a common constituent of the human microbiota; however, it can become pathogenic and cause infections in both immunocompetent and immunocompromised people. *C. albicans* exhibits remarkable metabolic versatility as it can colonize multiple body sites as a commensal or pathogen. Understanding how *C. albicans* adapts metabolically to each ecological niche is essential for developing novel therapeutic approaches. Purine metabolism has been targeted pharmaceutically in several diseases; however, the regulation of this pathway has not been fully elucidated in *C. albicans*. Here, we report how *C. albicans* controls the AMP *de novo* biosynthesis pathway in response to purine availability. We show that the lack of the transcription factors Grf10 and Bas1 leads to purine metabolic dysfunction, and this dysfunction affects the ability of *C. albicans* to establish infections.

## INTRODUCTION

*Candida albicans* is part of the human microbiota that resides harmlessly in the body; the immune system and other microbial communities play important roles by protecting the host from *C. albicans* overgrowth and tissue invasion (1, 2). However, *C. albicans* is an opportunistic pathogen and can become virulent in people with compromised immune systems. *C. albicans* causes a range of conditions from superficial infections in the epithelial mucosa to life-threatening bloodstream infections. To survive and cause infections in these diverse niches, *C. albicans* displays remarkable morphology reprogramming and metabolic adaptation. Morphological switching between yeast and filamentous forms is strongly associated with virulence (3–6). Transcriptomic analyses of strains with mutations in transcription factors (TFs) such as Gcn4, Tup1, Efg1, and Ace2 have revealed unexpected links between metabolism and virulence in *C. albicans* (7–11). These studies demonstrate that TFs coordinate the expression of genes related to both metabolism and virulence to ensure appropriate expression in particular microenvironments (12). However, it is still unclear if there are other TFs that regulate target genes in a similar manner and how TFs mechanically link metabolism and morphogenesis.

We recently reported on a role for the Grf10 homeodomain TF in regulating *C. albicans* morphogenesis (13). The *grf10*Δ mutant shows dramatically decreased hyphal growth on solid medium and a delay in germ tube formation in liquid medium. In addition, processes related to filamentation are strongly affected in the *grf10*Δ mutant, including an inability to generate chlamydospores, decreased biofilm formation, and attenuated virulence in mouse models of systemic infection (13, 14). Overexpression of *GRF10* triggers filamentation under conditions that normally promote yeast growth (15). Consistent with a role for Grf10 in morphogenesis, expression of *GRF10* is highly induced during filamentation (13), and *GRF10* is one of eight core target genes upregulated by the biofilm master regulators (16). Together, these results show that Grf10 is a critical TF that regulates filamentation and morphology-related traits in *C. albicans*.

The ortholog of *GRF10* from *Saccharomyces cerevisiae*, *PHO2* (*ScPHO2*), plays an important role in regulating metabolism. *Sc*Pho2 upregulates genes for purine biosynthesis, one-carbon metabolism, and histidine biosynthesis with the coregulator *Sc*Bas1, and it upregulates genes for acquisition and storage of inorganic phosphate with the coregulator *Sc*Pho4 (17–19). In *C. albicans*, *grf10*Δ (referred to as *pho2*Δ) and *bas1*Δ mutants exhibit a leaky adenine auxotrophy, whereas *pho4*Δ but not *grf10*Δ mutants exhibit a growth defect under phosphate limitation conditions (20), indicating a divergence of function. Importantly, purine biosynthetic genes have been shown to be involved with virulence in *C. albicans* (21–23), underlying a critical role of this metabolic pathway in fungal pathogenicity. Although maintenance of purine nucleotide pools is crucial for cell survival and pathogenicity, the genetic regulation of this pathway has not been well characterized in *C. albicans*.

Given the role for Grf10 in filamentation and virulence (13) and the observation that transcription factors coordinate regulation of virulence and metabolic genes (12), we investigated transcriptional regulation by Grf10. DNA microarray analysis was used to identify genes whose expression was dependent upon Grf10; genes for adenylate biosynthesis and also in diverse pathways such as iron homeostasis, one-carbon metabolism, adhesion, and other metabolic pathways were uncovered. The *bas1*Δ mutant had a lower growth rate than the *grf10*Δ strain in medium lacking adenine (−Ade). Using quantitative real-time PCR (qRT-PCR), the gene expression patterns of the wild type (WT), *grf10*Δ, and *bas1*Δ strains were determined in response to adenine limitation. Consistent with the DNA microarray data and growth phenotype, the *bas1*Δ and *grf10*Δ strains failed to derepress the *ADE* regulon and one-carbon metabolic genes, and the *bas1*Δ mutant showed a stronger *ADE* gene regulation defect than the *grf10*Δ mutant. *BAS1* plays an important role in pathogenicity, as the mutant exhibited attenuated virulence, although weaker than that of the *grf10*Δ mutant (13).

## RESULTS

### Identification of potential Grf10 target genes.

To characterize the global Grf10 target genes under yeast growth conditions, we performed DNA microarray analysis and determined differential gene expression in the *grf10*Δ (RAC117) mutant versus a *GRF10* strain (DAY185). Strains were grown at 30°C to the mid-log phase in synthetic dextrose (SD) medium with minimal supplements. The potential Grf10 targets were defined as those genes, out of 7,860 potential loci, in which expression was altered 2-fold or greater with a *P* value of 1 × 10^−5^ or lower. Our results revealed 25 genes that showed lower expression levels and 36 genes that showed higher expression levels in the *grf10*Δ mutant (Table 1; see Table S1 in the supplemental material). The genes that were differentially expressed in the *grf10*Δ mutant were sorted by the web-based GO Term Finder tool available on the *Candida* Genome Database (24) and manually sorted for uncharacterized genes.

10.1128/mSphere.00161-17.2 TABLE S1  Genes that changed expression in the *grf10* mutant by 2-fold or more and *P* values of 1 × 10^−5^. Download TABLE S1, XLSX file, 0.1 MB. Copyright © 2017 Wangsanut et al. 2017 Wangsanut et al. This content is distributed under the terms of the Creative Commons Attribution 4.0 International license.

**TABLE 1:** List of genes that are differentially expressed in the *grf10*Δ mutant

Group/GO term	FungiDB ID no.	Gene name	Function	Fold change	*P* value
Downregulated genes					
Purine metabolism (GO ID no. 6189, 46040, 6188, 72522, 9127)	orf19.3870	*ADE13*	Adenylosuccinate lyase	−4.14	4.48E−7
	orf19.7484	*ADE1*	Phosphoribosylaminoimadazole succinocarboxamide synthetase	−3.06	4.66E−8
	orf19.492	*ADE17*	5-Aminoimidazole-4-carboxamide ribotide transformylase	−2.85	8.01E−7
	orf19.5061	*ADE5,7*	Phosphoribosylamine-glycine ligase and phosphoribosylformylglycinamidine cyclo-ligase	−2.50	1.91E−6
	orf19.5906	*ADE2*	Phosphoribosylaminoimadazole carboxylase	−2.28	6.97E−6
	orf19.6317	*ADE6*	5-Phosphoribosylformyl glycinamidine synthetase	−1.97	9.74E−6
One-carbon metabolism	orf19.5750	*SHM2*	Cytoplasmic serine hydroxymethyltransferase	−3.48	1.50E−8
	orf19.5838	*SER2*	Ortholog(s) has phosphoserine phosphatase activity	−2.48	4.58E−6
	orf19.3810	*MTD1*	Ortholog(s) has methylenetetrahydrofolate dehydrogenase (NAD^+^) activity	−3.52	1.36E−6
	orf19.1117		Protein similar to *Candida boidinii* formate dehydrogenase	−3.69	5.00E−8
	orf19.1774		Predicted formate dehydrogenase	−4.51	8.99E−8
Iron metabolism	orf19.1932	*CFL4*	C terminus similar to ferric reductases	−3.89	1.12E−8
	orf19.1930	*CFL5*	Ferric reductase	−3.02	1.09E−6
Transcription	orf19.5338	*GAL4*	Zn(II)_2_ Cys6 transcription factor; involved in control of glycolysis	−2.03	4.66E−6
Miscellaneous	orf19.4025	*PRE1*	Putative β4 subunit of 20S proteasome	−40.42	1.23E−10
	orf19.4028	*RER2*	*cis*-Prenyltransferase involved in dolichol synthesis	−12.76	1.47E−8
	orf19.6570	*NUP*	Nucleoside permease; transports adenosine and guanosine	−7.15	1.65E−8
	orf19.1788	*XKS1*	Putative xylulokinase	−5.29	8.37E−9
	orf19.4024	*RIB5*	Putative riboflavin (vitamin B_2_) synthase	−4.48	7.32E−8
	orf19.4394		Protein of unknown function	−4.24	3.89E−8
	orf19.4814		Uncharacterized	−2.96	2.15E−7
	orf19.3222		Predicted vacuolar protein	−2.62	4.46E−7
	orf19.300	*AIP2*	Putative actin-interacting protein; *S. cerevisiae* ortholog is d-lactate dehydrogenase	−2.41	8.58E−8
	orf19.4441		Ortholog(s) involved in initiation of DNA replication	−2.16	2.97E−7
	orf19.1344		Protein of unknown function	−2.10	2.54E−6

Upregulated genes					
Cell adhesion and biofilm formation (GO ID no. 7155, 22610, 42710, 44011, 51703)	orf19.3548.1	*WH11*	White-phase yeast transcript	10.64	1.07E−8
	orf19.3160	*HSP12*	Heat shock protein	3.85	1.48E−7
	orf19.4216		Putative heat shock protein	3.64	1.88E−7
	orf19.2121	*ALS2*	ALS family protein; role in adhesion and biofilm formation	3.64	1.70E−6
	orf19.4555	*ALS4*	Glycosylphosphatidylinositol-anchored adhesin; roles in adhesion and germ tube induction	3.47	2.37E−7
	orf19.5437	*RHR2*	Glycerol 3-phosphatase; roles in osmotic tolerance	3.11	3.16E−6
	orf19.508	*QDR1*	Putative antibiotic resistance transporter	2.19	1.34E−5
	orf19.4477	*CSH1*	Aldo-keto reductase; role in fibronectin adhesion and cell surface hydrophobicity	2.11	2.51E−5
	orf19.1258		Adhesin-like protein	2.35	2.15E−6
	orf19.2475	*PGA26*	GPI-anchored adhesin-like protein of cell wall; role in cell wall integrity	2.00	2.36E−6
Miscellaneous	orf19.1868	*RNR22*	Putative ribonucleoside-diphosphate reductase	4.27	1.10E−7
	orf19.2531	*CSP37*	Cell wall protein, stationary-phase enriched, GlcNAc-induced	3.58	9.37E−8
	orf19.2633.1		Uncharacterized	3.23	9.14E−8
	orf19.1847	*ARO10*	Aromatic decarboxylase; catabolic alcohol synthesis	3.04	1.65E−7
	orf19.2048		Protein of unknown function	3.00	1.25E−6
	orf19.1863		Predicted Rho guanyl-nucleotide exchange factor activity	2.69	1.34E−5
	orf19.842	*ASR3*	Adenylyl cyclase and stress-responsive protein	2.50	4.13E−8
	orf19.3803	*MNN22*	α-1,2-Mannosyltransferase	2.50	2.30E−7
	orf19.125	*EBP1*	NADPH oxidoreductase	2.49	1.30E−6
	orf19.1862		Possible stress protein	2.36	1.51E−5
	orf19.251	*GLX3*	Glutathione-independent glyoxalase	2.36	2.52E−6
	orf19.3061.1		Ortholog of *S. cerevisiae* proteins Rps22A and Rps22B	2.32	1.22E−5
	orf19.5620		Protein of unknown function	2.24	1.38E−6
	orf19.1473		2-Hydroxyacid dehydrogenase domain-containing protein	2.20	2.85E−5
	orf19.1149	*MRF1*	Putative mitochondrial respiratory protein	2.19	2.69E−6
	orf19.4003	*TIP20*	Possibly involved in retrograde transport between Golgi apparatus and endoplasmic reticulum	2.01	3.78E−6
	orf19.1152		Protein of unknown function; induced in core stress response	2.01	1.06E−5
	orf19.6816		Putative xylose and arabinose reductase	2.00	5.98E−7
	orf19.2769		Putative protease B inhibitor	2.00	3.17E−6
	orf19.2047		Putative protein of unknown function; Hap43p-repressed gene	2.00	1.04E−5
	orf19.1691		*S. cerevisiae* ortholog is cytochrome *c* oxidase subunit	2.00	1.54E−5

**FIG 1:**
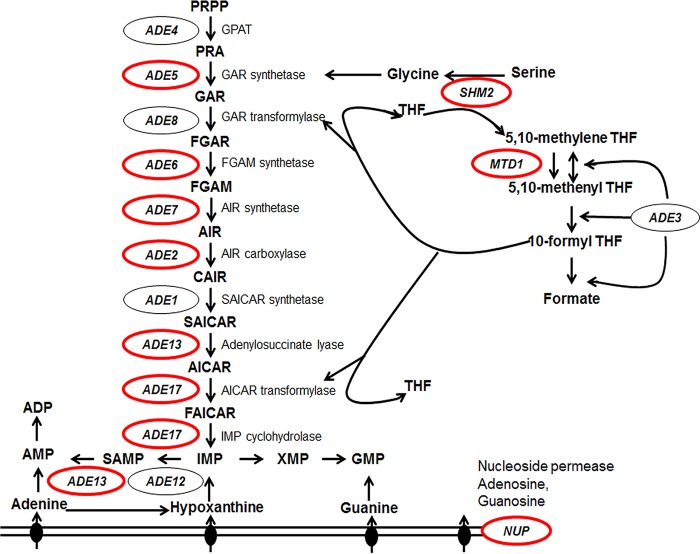
The purine salvage, AMP *de novo* biosynthesis, and one-carbon metabolic pathways of *C. albicans*. Genes identified by microarray analysis are circled in red. Enzymes and the genes encoding them that catalyze *de novo* purine biosynthesis and salvage pathways are as follows (from top to bottom): glutamine phosphoribosylpyrophosphate amidotransferase, *ADE4*; glycinamide ribotide synthase, *ADE5*; glycinamide ribotide transformylase, *ADE8*; formylglycinamide synthase, *ADE6*; aminoimidazole ribotide synthase, *ADE7*; aminoimidazole ribotide carboxylase, *ADE2*; succinylaminoimidazolecarboxamide ribotide synthase, *ADE1*; adenylosuccinate lyase, *ADE13*; aminoimidazole carboxamide ribotide transformylase and IMP cyclohydrolase, *ADE17*; adenylosuccinate synthase, *ADE12*; and nucleoside permease, *NUP*. Enzymes catalyzing the reactions in one-carbon metabolism and the genes that encode them are as follows: serine hydroxymethyltransferase, *SHM2*; NAD^+^-dependent 5,10-methylenetetrahydrafolate dehydrogenase, *MTD1*; mitochondrial C1-tetrahydrofolate synthase, *ADE3* (*MIS11*). Intermediate metabolites are abbreviated as follows (from top to bottom): PRPP, 5-phosphoribosyl-α-1-pyrophosphate; PRA, 5-phospho-β-d-ribosylamine; GAR, 5-phosphoribosyl-glycinamide; FGAR, 5′-phosphoribosyl-*N*-formylglycinamide; FGAM, 5′-phosphoribosyl-*N*-formylglycinamidine; AIR, 5′-phosphoribosyl-5-aminoimidazole; CAIR, 5′-phosphoribosyl-5-amino-imidazole-4-carboxylate; SAICAR, 5-amino-4-imidazole-*N*-succinocarboxamide ribonucleotide; AICAR, 5-amino-4-imidazolecarboxamide ribonucleotide; FAICAR, 5′-phosphoribosyl-4-carboxamide-5-formamidoimidazole; IMP, inosine monophosphate; SAMP, adenylosuccinate; THF, tetrahydrofolate.

Among the differentially expressed genes in the *grf10*Δ mutant, we found that most of the genes necessary for *de novo* adenylate synthesis—*ADE2*, *ADE5,7*, *ADE6*, *ADE13*, and *ADE17*—were strongly downregulated (Fig. 1; see Table S2 in the supplemental material). Additionally, we detected a decrease in expression of genes involved with the one-carbon metabolic pathway, which supplies the substrates glycine and N^10^-formyl tetrahydrofolate into the purine biosynthetic pathway (25). This gene set included *MTD1*, *SHM2*, *SER2*, and putative formate dehydrogenase-encoding genes orf19.1774 and orf19.1117. Additionally, we found lower expression of a gene encoding a nucleoside permease (*NUP* [orf19.6570]) potentially capable of transporting adenosine and guanosine (26). These gene products are all involved in the *de novo* biosynthesis, uptake, and interconversion pathways for purine nucleotides and account for 11 of the 25 downregulated genes. These results suggest that Grf10 is likely to directly regulate genes in purine *de novo* biosynthesis and related pathways.

10.1128/mSphere.00161-17.3 TABLE S2  GO term analysis of downregulated genes. Download TABLE S2, XLSX file, 0.1 MB. Copyright © 2017 Wangsanut et al. 2017 Wangsanut et al. This content is distributed under the terms of the Creative Commons Attribution 4.0 International license.

Other downregulated genes in the *grf10*Δ strain were in diverse pathways. The uncharacterized gene *PRE1*, which encodes the putative β4 subunit of the 20S proteasome, showed the greatest decrease in gene expression (~40-fold change) (Table 1; Table S2); ubiquitination and protein degradation have been implicated in metabolic adaptation and virulence (27). Gene *RER2* was downregulated 13-fold; *RER2* encodes *cis*-prenyltransferase, responsible for protein glycosylation, cell wall integrity, and cell separation (28). The *CFL4* and *CFL5* genes exhibit ~3- to 4-fold lower expression; these genes encode putative ferric reductases that may play a crucial role in iron homeostasis and virulence in *C. albicans* (29, 30). *RIB5*, which encodes a putative riboflavin synthase, is downregulated ~4.5-fold in the mutant, and *GAL4*, which encodes a transcription factor that regulates glycolysis (31, 32), was expressed ~2-fold lower.

The majority of the upregulated genes are involved in the cellular stress response, cell adhesion, and metabolic pathways (see Table S3 in the supplemental material). *WH11* shows the greatest increase in gene expression (~10-fold) in the *grf10*Δ mutant. *WH11* encodes a protein of unknown function but is homologous to *HSP12* of *S. cerevisiae*; it is expressed specifically in white-phase yeast cells, and its transcript is absent in hyphal cells (33). Several genes that had been identified as the core stress response genes, including *RHR2*, *HSP12*, and *GLX3*, are highly upregulated in the in the *grf10*Δ mutant (34, 35). Two members of the *ALS* gene family, *ALS2* and *ALS4*, which function in cell adhesion and biofilm formation (36), are upregulated ~3.5-fold, and *MNN22*, which encodes an α-1,2-mannosyltransferase that participates in cell wall biosynthesis (37), was expressed 2.5-fold higher. The *RNR22* gene, which encodes a putative ribonucleoside-diphosphate reductase, was expressed ~4-fold higher in the *grf10*Δ mutant. Finally, Gene Ontology (GO) term analysis with the GO Term Finder identified genes associated with carbohydrate metabolism, including d-xylose, arabinose, and pentose catabolic processes (Table S3) (*P* < 0.05). To summarize, the upregulated genes are involved in a range of biological processes.

10.1128/mSphere.00161-17.4 TABLE S3  GO term analysis of upregulated genes. Download TABLE S3, XLSX file, 0.1 MB. Copyright © 2017 Wangsanut et al. 2017 Wangsanut et al. This content is distributed under the terms of the Creative Commons Attribution 4.0 International license.

### The *grf10*Δ and *bas1*Δ mutants exhibit a growth defect in response to adenine limitation.

**FIG 2:**
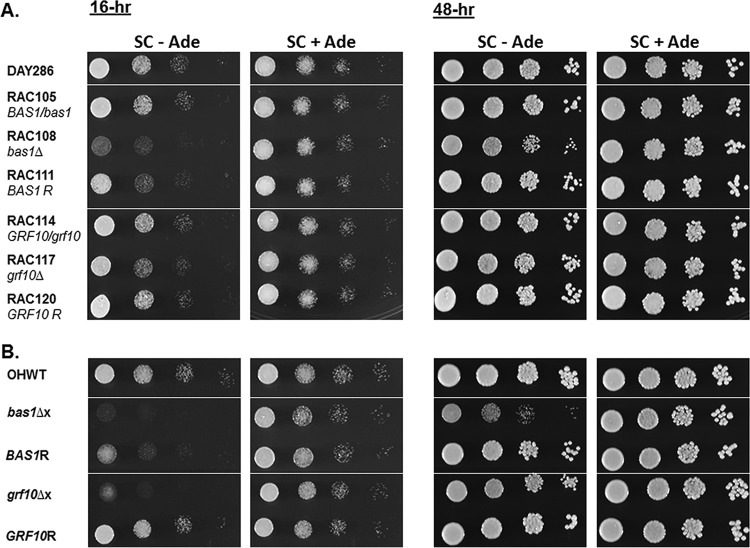
The *bas1*Δ and *grf10*Δ mutants exhibit leaky adenine auxotrophy. (A) The wild-type (DAY286), *BAS1* heterozygote (RAC105), *bas1*Δ (RAC108), and *BAS1* complemented (RAC111) strains and *GRF10* heterozygote (RAC114), *grf10*Δ (RAC117), and *GRF10* complemented (RAC120) strains from *C. albicans* in the BWP17 background were grown overnight in YPD medium and were spotted at a starting OD_600_ of 0.1 on plates containing SC agar medium with (+Ade) or without (−Ade) adenine. The plates were incubated for 48 h at 30°C. (B) The wild-type strain (OHWT), *bas1*Δ (TF016) and *grf10*Δ (TF021) mutant strains, and complemented (*BAS1R* and *GRF10R*) strains in the SN152 background were grown under the same conditions as in panel A.

In *S. cerevisiae*, *Sc*Pho2 interacts with *Sc*Bas1 to regulate adenylate and one-carbon metabolic genes (reviewed in reference 19). We examined the regulation of these genes under adenine limitation by Grf10 and its predicted protein partner Bas1 in *C. albicans*. We disrupted *BAS1* and *GRF10* genes in *C. albicans* strain BWP17 and also assayed the *bas1*Δ (TF016) and *grf10*Δ (TF021) mutants from the SN152 background (20) since strain background can influence phenotype. The slow growth of the null mutant strains on solid synthetic complete (SC) medium lacking adenine (SC−Ade) was more evident at 16 h than at 48 h, was more pronounced in the *bas1*Δ mutants than in the *grf10*Δ mutants, and was stronger in the SN152 background than in the BWP17 background (Fig. 2). By 48 h, only the *bas1*Δ strain exhibited slower growth than the parental wild-type strain; the *bas1* heterozygotes and all of the *grf10* mutants (heterozygotes and null) in both strain backgrounds were indistinguishable from their isogenic wild-type strains (Fig. 2). The adenine auxotrophy shown in Fig. 2 was strongest when cells received more nutrients, with growth in SC medium, than when we used SD medium (data not shown). We found that the adenine auxotrophic phenotype in *C. albicans* was weaker than that detected in the mutants from *S. cerevisiae* (data not shown).

**FIG 3:**
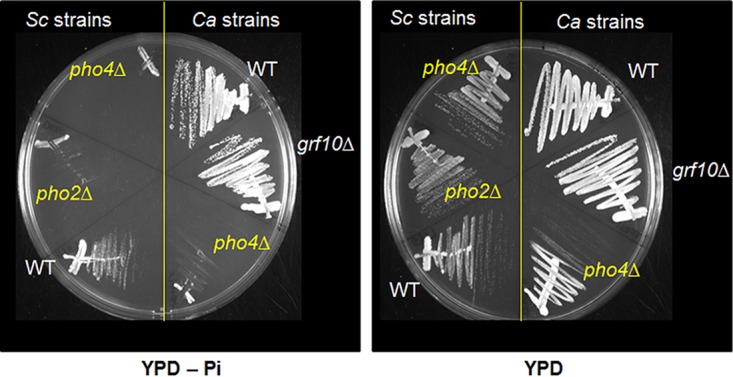
Grf10 is not required for growth under phosphate limitation in *C. albicans*. The *C. albicans* wild-type (OHWT) and *grf10*Δ (TF021Δ) and *pho4*Δ (TF004Δ) mutant strains and the control *S. cerevisiae* wild-type and *pho2*Δ and *pho4*Δ mutant strains were grown overnight in YPD medium and streaked out for single colonies on YPD medium with adenine containing or lacking (−Pi) inorganic phosphate for 2 days at 30°C.

In *S. cerevisiae*, *Sc*Pho2 interacts with *Sc*Pho4 to activate expression of genes encoding secreted acid phosphatases and phosphate transporters (19). To determine if Grf10 is important for the upregulation of *PHO* genes, the ability of the *Candida grf10*Δ mutant to grow on YPD medium containing 0.2 mM adenine and lacking inorganic phosphate was assessed. The *grf10*Δ mutant was able to grow without inorganic phosphate supplementation in the SN152 strain background (Fig. 3), as well as in the BWP17 background (data not shown). These findings contrast with the inability of both the *C. albicans pho4*Δ and *S. cerevisiae pho2*Δ mutants to grow, indicating that Grf10 is not required under phosphate starvation in *C. albicans*.

We tested a broad range of conditions to look for additional phenotypes, comparing the *bas1*Δ (RAC108) and *grf10*Δ (RAC117) strains with BWP17. Neither of the null mutations led to sensitivity to temperature (37, 40, and 45°C), cations (NaCl, KCl, and LiCl_2_), pH (range from pH 4.0 to 9.0), or oxidative stress (hydrogen peroxide, *t-*butyl hydroperoxide, and menadione).

To quantify adenine auxotrophy, we measured growth rates in liquid SC medium containing (+Ade) and lacking (−Ade) adenine. In the absence of adenine, we found that the *bas1*Δ mutants took nearly twice as long to grow as their respective WT strains (Table 2). In the *grf10*Δ mutants, there was only an ~10% increase in doubling times in both stain backgrounds. Restoring an allele of *BAS1* or *GRF10* to the null mutants complemented the growth defect—partially for *BAS1* and fully for *GRF10* (Table 2). In the presence of adenine, all of the mutants grew at the same rate as the wild type. Overall, the *bas1*Δ mutants showed stronger growth defects than the *grf10*Δ mutants in response to the absence of adenine. These results quantify the extent of the slow growth and demonstrate that the transcription factors differentially affect the Ade^−^ phenotype.

**TABLE 2:** Doubling times for the wild-type and *bas1*Δ and *grf10*Δ mutant strains

Strain	Doubling time (min)		Ratio
	+Ade	−Ade	
DAY286 (WT)	107 ± 1	104 ± 4	0.97
*BAS1*/*bas1*Δ mutant	106 ± 1	103 ± 1	0.97
*bas1*Δ mutant	107 ± 1	202 ± 6	1.89
*BAS1* restored	109 ± 4	140 ± 2	1.28
*GRF10*/*grf10*Δ mutant	108 ± 1	103 ± 1	0.95
*grf10*Δ mutant	104 ± 2	118 ± 1	1.13
*GRF10* restored	104 ± 2	103 ± 4	0.99
			
OHWT (WT)	107 ± 2	106 ± 1	0.99
*bas1*Δ mutant	110 ± 2	217 ± 5	1.97
*BAS1* restored	106 ± 2	143 ± 1	1.35
*grf10*Δ mutant	107 ± 4	118 ± 2	1.10
*GRF10* restored	107 ± 2	106 ± 3	0.99

### Bas1 and Grf10 upregulate the adenylate and one-carbon metabolic genes.

To examine transcription promoted by Grf10 and Bas1, we performed qRT-PCR to detect the expression of the nine *ADE* genes that constitute the adenylate biosynthetic pathway (Fig. 1). The wild-type, *bas1*Δ, and *grf10*Δ strains were grown at 30°C in SC medium with adenine and then shifted to medium lacking adenine, and cells were collected after 15 min for RNA preparation. *ADE* gene expression was compared with *TEF1* using the threshold cycle (Δ*C*_*T*_) method, and normalized to the expression in the wild-type strain under repressing (+Ade) conditions.

**FIG 4:**
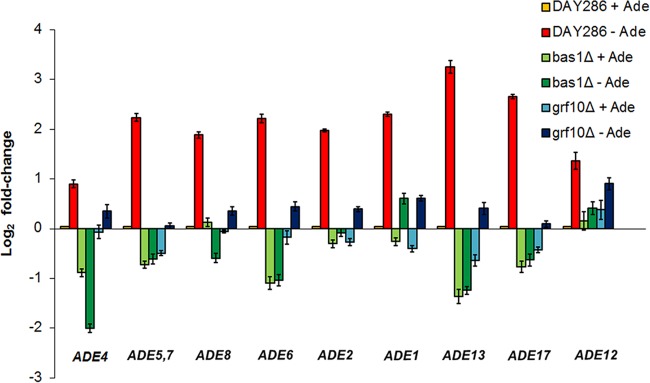
The expression of the *ADE* regulon is strongly downregulated in the *bas1*Δ and *grf10*Δ mutants. The wild-type strain (DAY286) and *bas1*Δ (RAC108) and *grf10*Δ (RAC117) mutant strains were grown in SC+Ade and shifted into media containing (+Ade) or lacking (−Ade) adenine; cells were harvested and RNA was prepared (see Materials and Methods for details). Relative gene expression was calculated by the Δ*C*_*T*_ method using *TEF1* as the reference gene. Expression was normalized to the wild-type strain under repressing (+Ade) conditions; this value is 0, but is depicted here as 0.1 for visualization (yellow bars). Elevated expression from the derepressed wild-type strain (−Ade) is shown as red bars; repressed and depressed expression levels from the *bas1*Δ mutant are shown in light green and dark green, respectively, and those for the *grf10*Δ mutant are shown in light blue and dark blue, respectively. Error bars indicate the standard deviation.

*ADE* genes were derepressed by 2- to 9-fold in the WT strain under adenine-limiting conditions (Fig. 4). Deletion of either Bas1 or Grf10 led to decreased expression of every *ADE* gene under adenine-limiting conditions, indicating that both Bas1 and Grf10 are required to achieve full expression. Bas1 appears to play an important role in maintaining basal expression, because the expression of several of the genes (*ADE4*, *ADE6*, and *ADE13*) was reduced by 2-fold or more in the *bas1*Δ strain relative to the WT under repressing (+Ade) conditions; however, basal expression of the *ADE* genes was not affected in the *grf10*Δ mutant. We examined the expression of *ADE13* in the heterozygous strains to determine if there was a dosage effect. Expression of *ADE13* in the *bas1* (RAC105) and *grf10* (RAC114) heterozygous mutants was not different from that in the wild-type strain DAY286 (see Fig. S1 in the supplemental material). Expression of *ADE13* was partially or fully restored when *BAS1* (RAC111) or *GRF10* (RAC120), respectively, was restored to the genome, consistent with the growth phenotypes shown in Fig. 2 and Table 2.

10.1128/mSphere.00161-17.1 FIG S1  Expression of *ADE13* in the *BAS1* and *GRF10* complemented strains. (A) Strains with mutations in *bas1*: wild type, DAY286; *BAS1*/*bas1*Δ heterozygote, RAC105; *bas1*Δ mutant, RAC108; *BAS1* restored strain, RAC111. (See Table 3 for the complete genotypes.) (B) Strains with mutations in *grf10*: wild type, DAY286; *GRF10*/*grf10*Δ heterozygote, RAC114; *grf10*Δ mutant, RAC117; and *GRF10* restored strain, RAC120. Strains were grown in SC+Ade and shifted into SC medium containing (+Ade) or lacking (−Ade) adenine; cells were harvested, and RNA was prepared (see Materials and Methods for details). Relative gene expression was calculated by the Δ*C*_*T*_ method using *TEF1* as the reference gene. Expression was normalized to the wild-type strain under repressing (+Ade) conditions; expression in DAY286 is repeated in panels A and B for ease of comparison. The repressed expression level from the wild type (+Ade) is shown as blue bars; elevated expression from the derepressed wild type (–Ade) is shown as red bars. Error bars indicate the standard deviation. *P* values were depicted and calculated by using Student’s *t* test function in Excel. *, *P* = 0.03; **, *P* ≤ 0.00025; NS, not significant. Download FIG S1, PDF file, 0.1 MB. Copyright © 2017 Wangsanut et al. 2017 Wangsanut et al. This content is distributed under the terms of the Creative Commons Attribution 4.0 International license.

**FIG 5:**
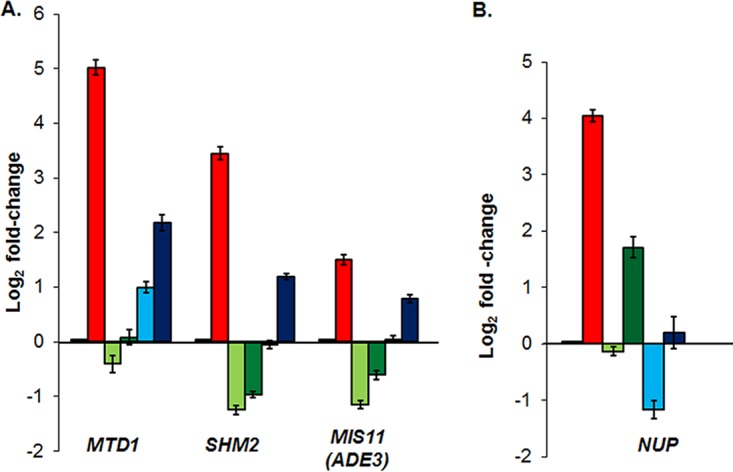
The expression of one-carbon metabolic genes and the expression of nucleoside permease are differentially regulated by Bas1 and Grf10. Strains were grown, RNA was prepared, and gene expression was analyzed as described in the legend to Fig. 4. The bars are color-coded as in Fig. 4. (A) Genes in the one-carbon metabolic pathway. (B) Expression of nucleoside permease. Error bars indicate the standard deviation.

Given the metabolic connection between adenylate and one-carbon metabolism and the results from the DNA microarray, we examined regulation patterns of one-carbon metabolic genes in *C. albicans*. We examined *MTD1*, *SHM2*, and *ADE3* (*MIS11*) by qRT-PCR as described above. When the wild-type strain was grown in medium lacking adenine, we detected the substantial upregulation of the one-carbon metabolic genes by 3- to 33-fold compared to basal expression in the WT strain (Fig. 5). This result indicates that adenine limitation leads to the coregulation of one-carbon metabolic genes. Because the expression of *SHM2* and *ADE3* genes in the *bas1*Δ strain was significantly below the WT levels (≥2-fold change), Bas1 was required for the basal expression of one-carbon metabolic genes. We found that basal expression of *MTD1*, *SHM2*, and *ADE3* (*MIS11*) genes is unaffected in the *grf10*Δ mutant.

Together, our results show that adenine limitation leads to cotranscriptional regulation of adenylate and one-carbon metabolic genes in *C. albicans*. Bas1 regulates both the basal and derepressed expression of *ADE* and one-carbon metabolic genes; however, Grf10 is necessary for the full upregulation of gene expression during derepression.

### Bas1 and Grf10 regulate *NUP* under the adenine derepressing conditions.

Our microarray data showed that the *NUP* gene, which encodes a nucleoside permease (26), was one of the genes most affected by the loss of Grf10. We reasoned that adenine limitation and both transcription factors Grf10 and Bas1 might also lead to the upregulation of this gene. We examined *NUP* gene expression by qRT-PCR as described above. The *NUP* gene was derepressed 17-fold in the WT strain grown in –Ade medium (Fig. 5). Both Bas1 and Grf10 were required for this transcriptional derepression; however, in contrast to the *ADE* and one-carbon metabolic genes, the basal and high-level expression of the *NUP* gene were more dependent on Grf10 than on Bas1. In the *grf10*Δ strain, *NUP* gene expression at basal levels (+Ade) was significantly below the WT levels (~2-fold change) and there was no derepression to high levels. However, in the *bas1*Δ strain, basal expression of *NUP* was not affected, and there was modest (3.4-fold) upregulation under –Ade conditions.

### Bas1 is implicated in virulence in an animal model of disseminated candidiasis.

**FIG 6:**
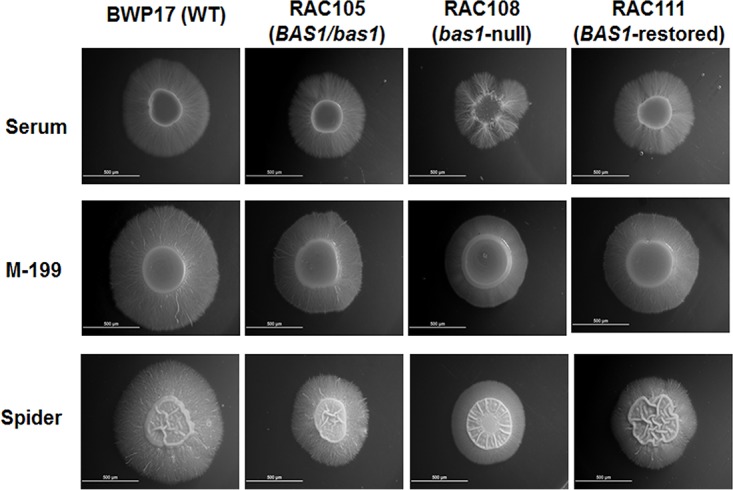
Disruption of *BAS1* mildly affects hyphal formation. To induce hyphae, wild-type and *bas1*Δ mutant strains were grown overnight in YPD medium and washed twice with sterile water. Cell densities were adjusted to 2 × 10^7^ CFU/ml, 5 µl of each strain was spotted onto YPD+10% serum, M-199, and Spider solid media, and plates were incubated for 3 days at 37°C and photographed. The induction of hyphae was performed at least three times, and representative examples are shown for each strain. Size bars, 500 μm.

Grf10 regulates morphogenesis and affects virulence (13, 15) in addition to regulating metabolic genes. This led us to examine the morphology and pathogenicity of the *bas1*Δ mutant. To investigate the role of Bas1 in morphology, we examined macroscopic colonies of the wild-type (BWP17), *bas1*Δ (RAC108), *BAS1* heterozygote (RAC105), and *BAS1* complemented (RAC111) strains under hypha-inducing conditions on solid M-199, Spider, and YPD medium containing 10% serum (Fig. 6), and compared these results with those reported for the *grf10*Δ mutants (13). On both solid M-199 and Spider media, mutations in *bas1* led to a decrease in the length of the filamentous region at the periphery of the colony, and on serum-containing medium, the *bas1*Δ colonies showed discontinuous hypha production in the periphery (Fig. 6). Addition of adenine to Spider medium did not alter this phenotypic difference between the null mutant and the wild type (data not shown). We also tested synthetic low-ammonia dextrose (SLAD) and SD+GlcNAc media, supplemented with and without adenine, for morphological differences; however, both the *bas1*Δ and *grf10*Δ mutants responded in the same manner as BWP17 to these hypha-inducing conditions (data not shown). Overall, the *bas1*Δ mutant exhibited mild morphological defects that are not as severe as those found in the *grf10*Δ mutant (13).

**FIG 7:**
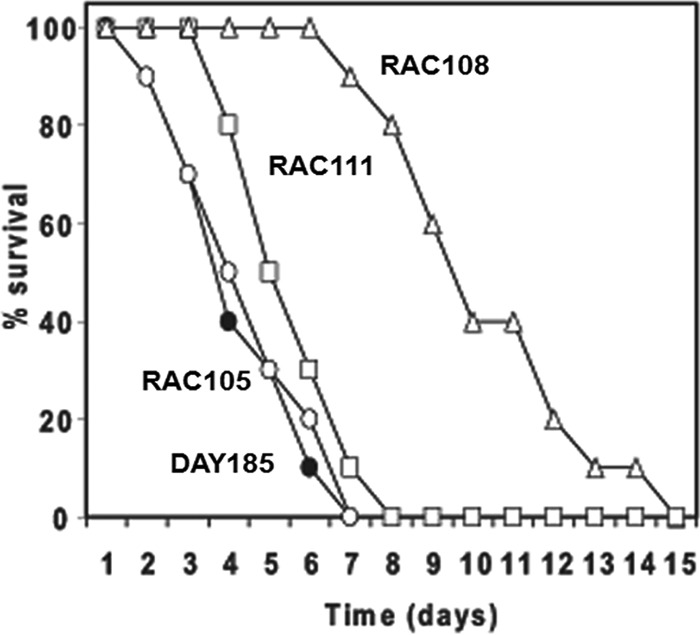
The *bas1*Δ mutant is less virulent in a mouse model of infection. Mice were infected with 1 × 10^6^ cells of a wild-type strain (DAY185) or with the heterozygous and homozygous null *BAS1* mutants through the lateral tail vein, and survival was monitored for up to 15 days postinfection. Shown is survival of mice infected with wild-type strain DAY185 (solid circles), the heterozygote strain RAC105 (open circles), the homozygous null mutant RAC108 (open triangles), or the restored strain RAC111 (open squares). Difference from DAY185 is significant for RAC108 (*P* = 7.53 × 10^−6^) but not significant for RAC105 and RAC111 (*P* > 0.05).

To assess the involvement of Bas1 in fungal virulence, we used a mouse model of disseminated candidiasis (38), comparing the survival rates of mice infected with the wild-type and *bas1* mutant strains. Mice infected with either of the two heterozygous *BAS1* mutants (RAC105 and RAC111) or the wild-type strain (DAY185) succumbed in 7 to 8 days (Fig. 7). The mice infected with the *bas1*Δ mutant (RAC108) survived longer than these, but all mice succumbed by about 2 weeks postinfection. The *bas1* null mutant was significantly different from the control strain (*P* = 7.53E−6), but neither the heterozygous mutant nor the restored strain was significantly different from the control (*P* > 0.05). We note that these strains differ in their auxotrophies for arginine and histidine; however, these differences are unlikely to have affected virulence because, first, the two heterozygotes RAC105 and RAC111 had similar virulence profiles in spite of their auxotrophic differences, and second, Noble and Johnson (39) found that neither the *arg4*Δ nor *his1*Δ mutation had an effect on virulence in the mouse systemic infection model. While ectopic *URA3* expression can affect virulence (40), this did not occur in this experiment because DAY185, RAC108, and RAC111 have the same virulence (Fig. 7), even though they differ in the location of *URA3* (at *ARG4* or *BAS1*). This finding indicates that the *bas1*Δ strain is attenuated for virulence in *C. albicans*.

## DISCUSSION

This study demonstrates that expression of nucleoside permease, adenylate biosynthetic, and one-carbon metabolic genes are transcriptionally regulated in *C. albicans* and that the Bas1 and Grf10 transcription factors are required for this regulation. The modulation of *ADE* gene expression by these transcription factors could potentially promote the survival of *C. albicans* in response to purine fluctuation in different sites on the human host. Bas1 plays a more critical role in the regulation of *ADE* and one-carbon metabolic genes than does Grf10. Indeed, the more severe gene expression defect in the *bas1*Δ mutant than in the *grf10*Δ mutant may explain its stronger growth defect (20) (Table 2; Fig. 4 and 5).

Our study indicates a largely but not wholly conserved role for the Bas1 and Grf10 orthologs of *C. albicans*. *C. albicans* Bas1 (*Ca*Bas1) shows conservation of gene targets with Bas1 from *S. cerevisiae* and *Ashbya gossypii*. One key difference is that *Ca*Bas1 regulates both basal and derepressed expression, whereas *Sc*Bas1 does not affect basal expression (19). The *A. gossypii* Bas1 (*Ag*Bas1) homolog controls genes for *de novo* purine biosynthesis as well as those in other metabolic pathways such as one-carbon metabolism and riboflavin biosynthesis (41). Another difference is that expression of *ADE3* and *NUP* is under the control of these factors in *C. albicans*. The *NUP* nucleoside permease transports adenosine and guanosine (26). There is no orthologue of *NUP* in *S. cerevisiae*; however, parasitic fungi and protozoa such as *Microsporidia*, *Leishmania*, *Trypanosoma*, *Trichomonas*, and *Plasmodium* require nucleoside permeases as they lack *de novo* purine biosynthesis (42–45). Although *C. albicans* is capable of synthesizing purines *de novo*, it may be possible that it upregulates the *NUP* gene to scavenge purine nucleosides.

It is striking that the adenine auxotrophy due to loss of Bas1 and Grf10 is weaker in *C. albicans* than it is in *S. cerevisiae*. This difference may reflect the different ecological niches for these species. *S. cerevisiae* is a generalist adapted to fruit (e.g., grapes) and to fermentation under anaerobic conditions (46). It can survive in a wide range of environments with various levels of nutrients, temperatures, osmolarities, and pHs; because of this, *S. cerevisiae* must tightly regulate gene expression to survive and respond to these diverse conditions (46, 47). *C. albicans* is adapted to the human host, colonizing different sites such as skin, mucosal tissue, and the bloodstream. Purine bases and nucleosides in plasma and extracellular fluids are found in low, virtually constant levels of ~4 μM (48, 49); high basal gene expression and feedback inhibition of the biosynthetic pathway could be sufficient to maintain intracellular nucleotide pools. In the intestine, there is intense competition among the microbial communities for nutrients released upon digestion. Nucleotidase in the small intestine hydrolyzes nucleotides to nucleosides (50), which would be available for uptake by nucleoside permease. It is interesting to speculate that the *NUP* gene of *Candida* may be particularly important for adaptation to this niche. Other infection sites may be more limited for purines.

Several reports demonstrate the crucial role of nucleotide biosynthesis for pathogens during infections. In *C. albicans*, mutants defective in purine or pyrimidine biosynthesis are avirulent during infections (51). Nucleotide biosynthesis is critical for the growth of bacterial pathogens such as *Escherichia coli*, *Salmonella enterica*, *Bacillus anthracis*, and *Staphylococcus aureus* in human blood serum or abscesses (52, 53). These reports strongly support the idea that pathogens commonly require nucleotide biosynthesis for growth during infection. Transcriptional upregulation in these niches may be crucial for full pathogenesis of *Candida*, accounting for the virulence attenuation in the *bas1*Δ (Fig. 7) and *grf10*Δ mutants (13).

Morphological changes in *C. albicans* from yeast to hyphal forms have long been linked to virulence, while emerging evidence shows that metabolic ability is also strongly linked to virulence (12). Morphological changes and metabolic adaptation are each controlled by complex transcriptional networks and are coordinated by transcription factors (12). Bas1 plays a prominent role in metabolism but only a marginal role in morphogenesis. However, Grf10 coregulates both virulence attributes (morphogenesis [13]) and fitness attributes (metabolism [this work]). We hypothesize that Grf10 regulates adenylate metabolism, morphogenesis, and other processes by interacting with different transcription factors. Future studies will shed light on how Grf10 coordinates fitness and virulence attributes.

## MATERIALS AND METHODS

### Yeast strains.

The strains of *C. albicans* used and generated in this study are listed in Table 3. Strains RAC114, RAC117, and RAC120 were described previously (13). Strains DAY185 and DAY286 were obtained from A. Mitchell (54), and strains SN152, OHWT, TF004Δ, TF016Δ, and TF021Δ (20) were obtained from the Fungal Genetics Stock Center. BWP17 (55) served as the parent strain for construction of the *BAS1* mutant strains (detailed below). Strains RAC255 and RAC256 carry restored alleles of *BAS1* and *GRF10*, respectively, in strains TF016Δ and TF021Δ (detailed below).

**TABLE 3:** Yeast strains used in this study

Strain	Relevant genotype	Reference or source
BWP17	*ura3*Δ::λ*imm434*/*ura3*Δ::λ*imm434 his1*::*hisG*/*his1*::*hisG arg4*::*hisG*/*arg4*::*hisG*	56
DAY185	*ura3*Δ::λ*imm434*/*ura3*Δ::λ*imm434 his1*::*hisG*::*HIS1*/*his1*::*hisG ARG4*::*URA3*::*arg4*::*hisG*/*arg4*::*hisG*	54
DAY286	*ura3*Δ::λ*imm434/ura3*Δ::λ*imm434 his1*::*hisG*/*his1*::*hisG ARG4*::*URA3*::*arg4*::*hisG*/*arg4*::*hisG*	54
RAC105	*ura3*Δ::λ*imm434*/*ura3*Δ::λ*imm434 arg4*::*hisG*/*arg4*::*hisG his1*::*hisG*/*his1*::*hisG BAS1*/*bas1*Δ::*URA3*	This study
RAC108	*ura3*Δ::λ*imm434/ura3*Δ::λ*imm434 arg4*::*hisG*/*arg4*::*hisG his1*::*hisG*/*his1*::*hisG bas1*Δ::*ARG4*/*bas1*Δ::*URA3*	This study
RAC111	*ura3*Δ::λ*imm434*/*ura3*Δ::λ*imm434 arg4*::*hisG*/*arg4*::*hisG his1*::*hisG*/*his1*::*hisG bas1*Δ::*ARG4*/*bas1*Δ::*URA3*::*<BAS1*, *HIS1>*	This study
RAC114	*ura3*Δ::λ*imm434*/*ura3*Δ::λ*imm434 arg4*::*hisG*/*arg4*::*hisG his1*::*hisG*/*his1*::*hisG GRF10*/*grf10*Δ::*URA3*	13
RAC117	*ura3*Δ::λ*imm434*/*ura3*Δ::λ*imm434 arg4*::*hisG*/*arg4*::*hisG his1*::*hisG*/*his1*::*hisG grf10*Δ::*ARG4*/*grf10*Δ::*URA3*	13
RAC120	*ura3*Δ::λ*imm434*/*ura3*Δ::λ*imm434 arg4*::*hisG*/*arg4*::*hisG his1*::*hisG*/*his1*::*hisG grf10*Δ::*ARG4*/*grf10*Δ::*URA3*::*<GRF10*, *HIS1>*	13
SN152	*arg4*Δ/*arg4*Δ *leu2*Δ/*leu2*Δ *his1*Δ/*his1*Δ *URA3*/*ura3*Δ *IRO1*/*iro1*Δ	39
OHWT	*arg4*Δ/*arg4*Δ::*ARG4 leu2*Δ/*leu2*Δ::*LEU2 his1*Δ/*his1*Δ::*HIS1 URA3*/*ura3*Δ *IRO1*/*iro1*Δ	20
TF016	*arg4*Δ/*arg4*Δ *leu2*Δ/*leu2*Δ *his1*Δ/*his1*Δ *URA3*/*ura3*Δ *IRO1*/*iro1*Δ *bas1*Δ::*HIS1*/*bas1*Δ::*LEU2*	20
RAC255	*arg4*Δ/*arg4*Δ *leu2*Δ/*leu2*Δ *his1*Δ/*his1*Δ *URA3*/*ura3*Δ *IRO1*/*iro1*Δ *bas1*Δ::*HIS1*/*bas1*Δ::*LEU2*::*<BAS1*, *SAT1* flipper*>*	This study
TF021	*arg4*Δ/*arg4*Δ *leu2*Δ/*leu2*Δ *his1*Δ/*his1*Δ *URA3*/*ura3*Δ *IRO1*/*iro1*Δ *grf10*Δ::*HIS1*/*grf10*Δ::*LEU2*	20
RAC256	*arg4*Δ/*arg4*Δ *leu2*Δ/*leu2*Δ *his1*Δ/*his1*Δ *URA3*/*ura3*Δ *IRO1*/*iro1*Δ *grf10*Δ::*HIS1*/*grf10*Δ::*LEU2*::*<GRF10*, *SAT1* flipper*>*	This study
TF004	*arg4*Δ/*arg4*Δ *leu2*Δ/*leu2*Δ *his1*Δ/*his1*Δ *URA3*/*ura3*Δ *IRO1*/*iro1*Δ *pho4*Δ::*HIS1*/*pho4*Δ::*LEU2*	20

PCR with pGEM-URA3 (56) and primers BAS1-5DR and BAS1-3DR (portion of the primer that anneals with the vector is shown in lowercase in Table 4) was used to generate a fragment carrying the *bas1*Δ::*URA3* allele. After transformation of BWP17, the deletion was confirmed in strain RAC105 using primers to *BAS1* and *URA3*. To generate the null mutant, the same primers and pRS-ARG4 were used to amplify a fragment carrying the *bas1*Δ::*ARG4* allele. This fragment was transformed into RAC105, and confirmation of the genotype in strain RAC108 was made by PCR and by Southern analysis (data not shown).

**TABLE 4:** Primers used in this study

Name	Description	Sequence (5′→3′)*^a^*
BAS1-5DR	*BAS1*-pGEM3 sequence	CCAAATCCTCTGATGGTTTTATGCAACCCAGATTATTTTAGCATTCTAACTCGTATCAGCgttttcccagtcacgacgtt
BAS1-3DR	*BAS1*-pGEM3 sequence	ACTACAATCAATCATCGTATATTCTTACATTAGCATCTGATTCTTATACACTAGAATACCtgtggaattgtgagcggata
BAS1-DF	Diagnostic forward primer	GTGAAGTTTCTGATGCGAC
BAS1-DR	Diagnostic reverse primer	GCCAAGGGACCTATTTGC
B1RF	Restored allele forward primer	CTGGATCCATTGGCAGCATTATTG
B1RR	Restored allele reverse primer	ACGGATCCACGCCTTAACCAACT
G10RF	Restored allele forward primer	AGTGGGCCCCTTAGTATTCAACGA
G10RR	Restored allele reverse primer	TGAGGGCCCGTATCATGACTTTG
*ADE1*	Forward primer	GAGACTATGCTGCTACTAAAGG
	Reverse primer	CAACACTTCGTCAACAAGAAC
*ADE2*	Forward primer	CGATTCGGATCTACCAGTTATG
	Reverse primer	GGAGTTCTGTGTGCACTTAC
*ADE4*	Forward primer	GTTGCCATGGCTAGAGAAG
	Reverse primer	TGGTGTCAGCTAAATCAATCC
*ADE5,7*	Forward primer	CTCATATTACTGGTGGAGGATTAG
	Reverse primer	ATCTCTGGTACTTGCCATTG
*ADE6*	Forward primer	GCAGCTGATATCCCTTCATTAG
	Reverse primer	TCCATACCAATGGCTTGAA
*ADE8*	Forward primer	CTTTGGAGAAGGCAGGAATC
	Reverse primer	CTCCATCTTGACCAGCTTTC
*ADE12*	Forward primer	GGTCCATTCCCAACAGAAC
	Reverse primer	ATCCAACCAACCACATCTTC
*ADE13*	Forward primer	ACAAGAAGGTGGCGATAATG
	Reverse primer	GTTTGTTGAGGAGCTCTACC
*ADE17*	Forward primer	AACAAGGTGCTGTTGATTTG
	Reverse primer	CTCCTAAGCCGATAACCATAC
*MTD1*	Forward primer	TGTCCCATCCATTGGTAAAG
	Reverse primer	AAGAGGTCGCATCAGAAAC
*SHM2*	Forward primer	CAAATTGATGGTGCTAGAGTTG
	Reverse primer	CTAACTCCACCTGGAACTAAAG
*MIS11* (*ADE3*)	Forward primer	AATGTATGGTGCTGGTGAAG
	Reverse primer	GTCTTGGCGATACAGATTGG
*NUP*	Forward primer	GACCACCTCCATCAATGTC
	Reverse primer	TTGGAGTACCAGCAATAACC
*TEF1*	Forward primer	TTCGTCAAATCCGGTGATG
	Reverse primer	CTGACAGCGAATCTACCTAATG

a Lowercase represents the nucleotides that anneal with the vector.

We introduced a functional allele of *BAS1* into two *bas1*Δ strains, RAC108 and TF016Δ (20). For restored strain RAC111, we amplified a 3.2-kb fragment carrying the native *BAS1* locus using primers B1RF and B1RR (Table 4). The fragment was inserted into the BamHI site of pGEM-HIS1, generating pGHBF. Plasmid pGHBF has a unique PshAI site located upstream of the *BAS1* gene; RAC108 was transformed with PshAI-cleaved pGHBF, selecting for histidine prototrophy. Integration of *BAS1*::*HIS1* into the *bas1*Δ::*URA3* allele was confirmed by PCR amplification. To generate restored strain RAC255, we subcloned the 3.2-kb BamHI fragment from plasmid pGHBF into the *SAT1* flipper-containing plasmid pSFS2 (57), generating plasmid pSFS2A-*BAS1*. TF016Δ was transformed with PshAI-cleaved pSFS2A-*BAS1* and selecting for nourseothricin resistance (58). Integration was confirmed by PCR amplification using primers to *BAS1* and within the *SAT1* flipper cassette.

We introduced a functional allele of *GRF10* into strain TF021Δ. *GRF10* was amplified using primers G10RF and G10RR (Table 4), and was inserted into the PspOMI site of pGEM-HIS1, generating pGHPF. The 2.8-kb PspOMI fragment from plasmid pGHBF was subcloned into the PspOMI of pSFS2 (57), generating pSFS2A-*GRF10*. This plasmid was cleaved by BglII and transformed into TF021Δ, selecting for nourseothricin resistance, generating strain RAC256. Integration was confirmed by PCR amplification of genomic DNA using primers to *GRF10* and within the *SAT1* flipper cassette.

### Media and growth conditions.

Strains were grown on yeast extract-peptone-dextrose (YPD) and synthetic dextrose (SD) medium (59) at 30°C. SD medium (2% dextrose, 6.7% yeast nitrogen base [YNB] plus ammonium sulfate) was supplemented with minimal supplements (0.5 mM uridine [Uri], 0.1 mM histidine, 0.1 mM arginine, and 0.15 mM adenine [Ade]). Synthetic complete (SC) medium was prepared by supplementing SD medium with CSM−Ade+Uri (Sunrise Science) (20). YPD medium containing 0.2 mM adenine was depleted for inorganic phosphate (YPD−P_i_) as described previously (60). Hypha formation was monitored on the following solid 1.5% agar media: Spider medium (61), 10% fetal calf serum, and M-199 (Gibco-BRL) buffered with 155 mM HEPES (pH 7.5). All strains were maintained at 4°C on YPD plates and cultured monthly from frozen stocks.

### Spot growth assay.

Strains were grown overnight in 5 ml YPD broth. The culture was diluted into sterile water to a starting optical density at 600 nm (OD_600_) of 0.1. The cultures were serially diluted 1:10 into sterile water; 3 μl of each dilution was spotted onto SC+Ade and SC–Ade plates. The plates were incubated at 30°C and photographed with an ImageQuant imager daily. Each strain was tested with at least three biological replicates.

### Growth rate and doubling time determination.

Strains were grown overnight at 30°C in 5 ml YPD broth and diluted 1:50 dilution into SC+Ade and SC–Ade media. Two 200-μl samples were transferred from each diluted culture into separate wells of a 96-well plate that was placed at 30°C in a thermo-controlled GloMax plate reader. The OD_600_ of each well was measured every 30 min over 24 h, shaking the plate for 30 s prior to each OD_600_ reading. Samples were standardized to wells containing sterile medium. *P* values and standard deviation were calculated using the *t* test and standard deviation functions in Excel. Each strain test was performed with three biological replicates.

### DNA microarray and data analysis.

Yeast strains DAY185 and RAC117 (*grf10*Δ null) were grown overnight in SD medium with minimal supplements and inoculated at a 1:50 dilution into 50 ml of fresh medium in triplicate. The cultures were grown until the mid-log phase (OD_600_ of ~0.5 to 1). The cells were quickly chilled in an ice-water bath and harvested by centrifugation, and RNA from three biological replicates was extracted using the RiboPure yeast kit, following the manufacturer’s instructions.

The gene profiling experiment was performed by ClinEuroDiag, Brussels, Belgium. Full genomic *C. albicans* DNA microarrays were developed and designed by the Galar Fungal Consortium and produced by ClinEuroDiag. Fluorescence-labeled cDNA was prepared from 1 μg of total RNA, using the Ambion amino allyl MessageAmp II aRNA postlabeling kit. After purification, both samples were combined and the volume was reduced. The labeled cDNA mix was resuspended in 60 µl hybridization buffer (ClinEuroDiag) and used for hybridization.

The microarrays were prehybridized at 42°C for at least 45 min. Afterwards slides were washed 5× with distilled water and spin-dried at 900 rpm at room temperature for 5 min. The labeled cDNA mixture was denatured for 2 min at 95°C prior to overnight hybridization (at least 16 h at 42°C) using the Advalytix hybridization station SB800 (Beckman Coulter, Inc.). The microarrays were washed for 5 min in 0.2× SSC–0.1% SDS with constant agitation at room temperature and rinsed for 5 min in 0.2× SSC at room temperature (1× SSC is 0.15 M NaCl plus 0.015 M sodium citrate). The microarrays were spin-dried at 900 rpm for 5 min. Afterwards the microarrays were scanned with the GenePix 4000B microarray scanner (Molecular Devices), and the signal intensities were analyzed using GenePix Pro 5.1 image acquisition and data analysis software (Molecular Devices).

Intensity-dependent normalization was performed by applying a locally weighted linear regression analysis (locally weighted scatterplot smoothing [LOWESS]). The data were calculated as a log of the signal intensities of the average of two identical spots (R1 and R2) for three biological replicates, and the *P* values were calculated. DNA microarray data were sorted based on the cutoff *P* value of <0.00001 and >2-fold change. Gene classification was based on the *C. albicans* GO term and performed using the GO Term Finder and Go Slim Mapper tools available at the *Candida* Genome Database website (candidagenome.org).

### qRT-PCR analysis.

Cells were grown and harvested as previously described (62). Briefly, yeast strains were grown overnight in liquid SC medium, inoculated 1:50 into 25 ml of fresh SC+Uri+Ade medium, and grown at 30°C until reaching the mid-log phase (OD_600_ of ~0.5 to 1). The mid-log-phase culture was split in half, pelleted for 2 min at room temperature, washed twice with prewarmed SC+Uri+Ade or SC+Uri–Ade, and then resuspended in the same medium. After 15 min of incubation at 30°C, 5 ml of each sample was removed and quickly chilled in an ice-water bath, and cells were harvested by pelleting for 2 min at 4°C. The cell pellet was immediately frozen on dry ice and stored at −80°C. Three biological replicates were harvested for each sample.

RNA was extracted from the frozen cell pellets and converted to cDNA as previously described (13). The RT-qPCR was performed in duplicate using the SensiFAST SYBR No-ROX kit, as described previously (13). Gene *TEF1* (*EFT3*) was included as a reference gene. All primers used in this study are listed in Table 4. The relative gene expression was calculated by the Δ*C*_*T*_ method using a reference gene as described by Bio-Rad Laboratories. The Student’s *t* test and statistical significance were calculated by using Excel.

### Virulence determination.

The determination of virulence of the *bas1*Δ strains RAC105, RAC108, and RAC111 was performed at the same time and in the same manner as for the *grf10*Δ strains that have been published (13). Briefly, *C. albicans* strains were grown in YPD broth at 30°C to stationary phase, washed twice with calcium- and magnesium-free phosphate-buffered saline (PBS), and resuspended in PBS at a cell density of 5 × 10^6^ cells·ml^−1^ based on hemocytometer counts. Virulence in mice was assessed as described previously (38, 63). Groups of 10 male BALB/c mice (body weight, 20 to 22 g [Harlan]) were formed, and each mouse was injected through the lateral tail vein with a 200-µl inoculum containing 10^6^ cells of wild-type control or mutant yeast. Mice were given food and water *ad libitum*. Survival of the mice was monitored twice daily, and moribund mice were euthanized by asphyxiation with carbon dioxide, as recommended by the American Veterinary Medical Association (64). Kaplan-Meier survival curves were created using SPSS 15.0 software; the survival curves were compared by the Mantel-Haenszel log-rank test as implemented in the package “survival” (65) for R (66).

### Accession number(s).

Microarray data are available at the ArrayExpress database under accession no. E-MTAB-5798.
