# Supplementary figures and images for: Grf10 and Bas1 Regulate Transcription of Adenylate and One-Carbon Biosynthesis Genes and Affect Virulence in the Human Fungal Pathogen Candida albicans
Source: mSphere. 2017 Aug 2;2(4):e00161-17. doi: 10.1128/mSphere.00161-17 (PMC5541157; doi:10.1128/mSphere.00161-17)

**A.**

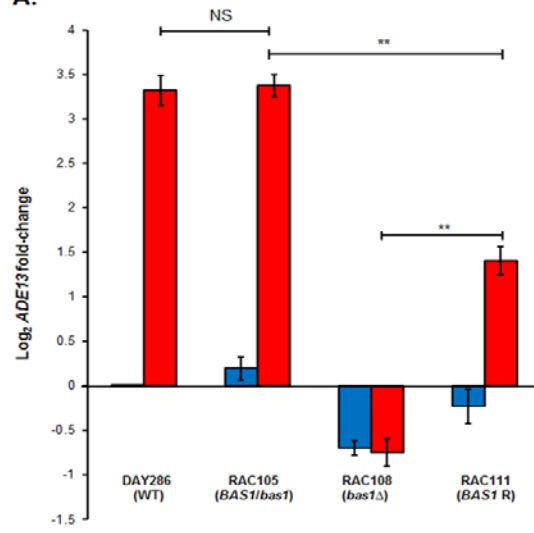

**B.**

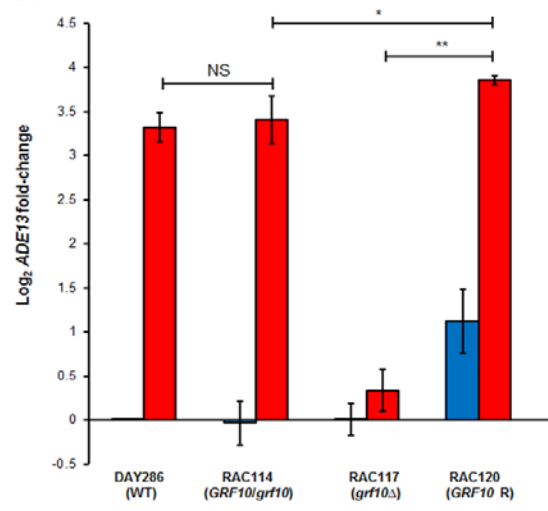

Supplement: FIG S1 [file sph004172329sf4.pdf]
